# Empowering Nurses Through Innovative Learning: A Path to Better Health and Care

**DOI:** 10.1002/hsr2.70268

**Published:** 2024-12-09

**Authors:** John Patrick C. Toledo

**Affiliations:** ^1^ Department of Theology and Religious Education De La Salle University Manila Philippines

Dear Editor,

The study titled “The importance of integrating flexible learning methods (audio‐visual animation vs. visual pamphlet) to enhance awareness, perspectives, and practices in preventing lower back pain in nurses” conducted by Nahid Zarifsanaiey, Zahra Yazdani, Zahra Karimian, and Hadi Raeisi Shahraki is an important study that looks into the teaching methods that can enhance nurses' healthcare practices.

This quasi‐experimental study aimed to compare the effectiveness of two different flexible learning methods—audio‐visual animation and visual pamphlets—on nurses’ awareness, perspectives, and practices regarding the prevention of lower back pain (LBP) [1]. According to the study's findings, both educational treatments considerably improved the participating nurses' knowledge and preventive behaviors; however, the audio‐visual animation group had the biggest gains in awareness and attitude regarding LBP prevention techniques.

This study has significant implications for high‐quality healthcare. Because of the severe physical demands of their jobs, nurses frequently have lower back discomfort, which can impair both their performance and the standard of patient care. This study shows that effective training tactics enable nurses to adopt better behaviors for their own well‐being, which in turn results in higher‐quality patient care. Nurses are less likely to sustain work‐related injuries that impair their capacity to give care when they know how to avoid lower back pain. Therefore, incorporating flexible learning strategies guarantees that patient care stays efficient and consistent while also promoting the health of nurses.

The study also emphasizes how important it is for nurses to continue their professional growth. Giving nurses the appropriate resources and information is essential in a time when healthcare has become more complicated. Adaptable teaching strategies, including audio‐visual aids, accommodate various learning preferences and make it simpler for nurses to interact with the content and use what they have learned in real‐world situations. Both patients and healthcare professionals may experience better health outcomes as a result of this greater involvement.

Given the current difficulties faced by Filipino nurses, the study's topics are especially relevant in the Philippine setting. Nurses, who make up a sizable section of the Philippine healthcare industry, frequently struggle with low pay, which results in job discontent and high turnover rates. According to reports, a large number of Filipino nurses are paid less than their counterparts in wealthy nations. The average monthly salary of nurses in the Philippines varies significantly, with those in the private sector earning around ₱12,000–₱15,000, while public sector nurses earn a base pay starting at ₱36,619 [2]. The gap highlights the stress and strain that nurses endure, which is exacerbated by inadequate facilities and resources, which may also impair their ability to deliver high‐quality treatment.

Including successful teaching strategies, like those covered in Zarifsanaiey's research, could have two uses. In addition to improving behaviors and expertise, it may help nurses feel more valued as professionals. Nurses may be more likely to stay in the field and lessen turnover and the resulting burden on healthcare systems if they believe their health and well‐being are valued through adequate training and resources.

All things considered, the study emphasizes how important adaptive instructional techniques are to enhancing nurses' healthcare practices. The research supports the health of nurses and their patients by addressing the prevention of lower back pain through flexible learning approaches. Such findings are crucial in promoting systemic changes that acknowledge and reward nurses' efforts, which will ultimately result in a more resilient and accessible healthcare system in a nation like the Philippines where low pay and unfavorable working circumstances are common.

## Author Contributions

**John Patrick C. Toledo:** conceptualization, methodology, writing, and submission.

## Ethics statement

It was a study of general information that was accessible online. There was no need for ethical approval.

## Conflicts of Interest

The author declares no conflicts of interest.

## Transparency Statement

The author, Mr. John P. C. Toledo, confirms that the study described in this publication is truthful, accurate, and transparent; that no significant details have been left out; and that any changes from the planned (and, if applicable) study have been discussed.

## Data Availability

The data that support the findings of this study are openly available at https://onlinelibrary.wiley.com/doi/10.1002/hsr2.70127.
